# 

**Published:** 1890-09-20

**Authors:** 


### NOTICE TO CORRESPONDENTS.

All MS., Letters, Books for Review, and other matters intended for the Editor should be addressed The Editor, The Lodge, Porchester Square, London, W.

The Editor cannot undertake to return rejected M.S., even when accompanied by stamped directed envelope.

### Notice to Advertisers and Subscribers.

All Advertisements, Orders for Copies of The Hospital, and Business Communications should be addressed to The Publisher, *not to the Editor*, The Hospital, 140, Strand, W.C.

### Bound Volumes of "The Hospital."

Vol. VI., containing full page portraits of T.R.H. the Prince and Princess of Wales, and Miss Florence Nightingale, is obtainable at 4s., post free.

Orders will be received for Vol. VII., 4s. post free. Cases for binding may be had of the Publisher, at 1s. 6d. each.

### To Residents, Secretaries, and Matrons.

The Editor will be glad to have the Regulations and each Report as issued by Asylums, Hospitals, Convalescent and Nursing Institutions, as well as those of other Charities, and an early copy in duplicate of all Appeals and Festival Notices, etc., addressed to *The Editor, The Lodge, Porchester Square, London, W.* Any superintendent, secretary, matron, or other chief official who regularly sends such information may be registered, on application to the Publisher, to receive free of cost a cover for binding The Hospital in half-yearly volumes.

### BURDETT'S HOSPITAL ANNUAL FOR 1890

**Is now ready, and may be obtained of the Publisher, The Hospital Offices, 140, Strand, W.C., price 2s. 6d. limp boards, or 3s. 6d. cloth. All orders must be accompanied by a remittance.**

### General Charities.

[This Column is devoted to an account of work of charitable institutions other than Medical Charities. The Editor will be glad to receive reports or news of work at 140, Strand. *Philanthropy* should be plainly written on the envelope.]

There is a practical idea suggested by a lady writer in the *Daily Graphic* which might be of use to those interested in the **PROVIDING EMPLOYMENT FOR WOMEN.** Writing on the subject of the immorality of cheapness she says that much of the evil of overworked girls arises from the mania of "ready made" clothes. Our grandparents employed a dressmaker who came by the day or week, and who received good food and adequate pay. We are quite sure that there is room for a number of such workers nowadays, but those there are, are, as a rule, not very competent, and besides they are anxious for too high pay. To be any use, such workers ought to be thoroughly trained, not possessing merely a smattering of their trade. This is employment which would not interfere with the independence of women, and is not independence the great craving of the present day? An excellent virtue when it is solid and genuine, but one, which is fast degenerating, in many cases, into an excuse for wilfulness.

**THE GORDON BOYS' HOME** sends us a report for August, 1890. The charity received in the past year £17,446. Of this sum £5,609 was expended in buildings and alterations, so that out of their large income the committee, after finding the general expenses, &c., were only able to invest £5,273. £4,000 of last year's money was contributed by the late Mr. Junius Morgan, who certainly was everybody's friend. £2,000 was the gift of Mr. G. W. Rusden. There are now 180 boys, ranging from 14 to 18 years of age, in the Home, which is situated at West End, Chobham, on the breezy common between Woking and Bagshot. Each boy on his entrance chooses his trade or profession, in which he receives a thoroughly practical training under skilled instructors. Carpenters, smiths, tailors, shoemakers, cooks, bakers, and musicians are all trained at this Home, so that there is plenty of choice afforded to the inmates. Whatever the employment, each boy attends school and religious instruction daily. The Roman Catholics are visited by a priest of their own faith. Sixteen boys have enlisted in the army, ten are earning livings in their trades, while five have entered domestic service. An excellent plan is that when the boys leave the Home, touch is kept with them as far as possible, and reports are received from regiments and employers regarding the characters they bear, and greatly to the praise of the Home, nearly all are satisfactory. The candidate for entrance to this institution must be really poor and unprovided for, he must not have any physical infirmity, also, he must not have been convicted of crime. There are the names of eminent men on the committee, and we can well understand the motives they had in making this rule of exception. But what comes under the heading of crime? It is a word capable of containing a vast amount of meaning. Though evil communications are corrupting, still, on the other hand, there is something to be said on behalf of the juvenile sinner who, if he be condemned to a reformatory, associates only with those whose moral natures are probably not an atom higher than his own, so that his chance of moral elevation is very small indeed. The difficulties of deciding whether we are to save the sinner or protect the saint are doubtless manifold, enough to perplex the mind of the ablest student of the human heart, but as we are the creatures of circumstances to an enormous extent, and our sympathies are strongly with the sinners who, given a helping hand and the example of better natures, would probably turn out fine honest men, whereas as things now stand they have no chance of moral progression at all. Gordon was one of the purest men, and this home would have been a great satisfaction to him, for the great hope of reformation lies in the work we achieve among children; the reformation of the adult is a far deeper problem. "The Gordon Boys' Home," an article by Lieut.-Colonel Collins, is full of interest. In it he says that Miss Gordon told him that her brother controlled the rough Chatham lads so well by his own personal interest. "For this reason," says the writer, "I think no individual home should be numerically unwieldy." And then again, "up to a certain ratio they (the incorrigible boys of any establishment) can be easily kept in hand, but should that number be exceeded, they become a very dangerous element, and will leaven the whole lump with insubordination and mischief." This mischief is what must attend all huge institutions, but the Gordon Boys' Home will, when finished, only accommodate 320. It is a national memorial and a work of most excellent merit, based on the knowledge that efficiency of technical and moral training to the boys is the great hope for future generations. All contributions (which are most earnestly asked for) to be sent to the secretary, Lieut.-Colonel G. A. Beaty-Pownall, 20, Cockspur-street, S.W.

**THE POOR CHILDREN'S AID SOCIETY** have handed the work of providing dinners over to the London Schools Dinners Association. A great part of the work of the Poor Children's Aid Society consists in the clothing loan department. The borrower signs a receipt for the clothes, and during the time of the society's operations there have only been two cases of abuse. Great endeavours have been made to always ascertain that the grants are made to genuine cases of distress. Miss Mary Winkfield, the Secretary and Lady Superintendent, will be very grateful for any gifts of clothing. Perhaps one of the best branches of the work is that the society helps crêches with gifts of clothing, and the poor London infants want aid more than anybody. Office, 34, Piccadilly Circus.

